# Posterior reversible encephalopathy syndrome and reversible cerebral vasoconstriction syndrome associated spinal subdural hematoma: A case report

**DOI:** 10.1097/MD.0000000000021522

**Published:** 2020-07-31

**Authors:** Hanfeng Chen, Ziqi Xu, Yuan Yuan

**Affiliations:** Department of Neurology, The First Affiliated Hospital, College of Medicine, Zhejiang University, Hangzhou, China.

**Keywords:** digital subtraction angiography, posterior reversible encephalopathy syndrome, reversible cerebral vasoconstriction syndrome, spinal subdural hematoma

## Abstract

**Rationale:**

Posterior reversible encephalopathy syndrome (PRES) and reversible cerebral vasoconstriction syndrome (RCVS) are separate clinical entities with distinct pathophysiological features. But in some special conditions PRES and RCVS can occur simultaneously.

**Patient concerns:**

We report the unique case of a 40-year-old female presented with crescendo headache, blurred vision, and recurrent generalized tonic–clonic seizure. She had a minor neck injury 1 week before but attracted no more attention. Neurological tests on admission yielded a Glasgow Coma Scale score of 13. No obvious focal neurological deficit apart from positive signs of meningeal irritation was presented.

**Diagnoses:**

Xanthochromia and hemorrhagic cerebrospinal fluid with pleocytosis was found on lumbar puncture. Cranial computed tomography was negative but magnetic resonance imaging demonstrated bilateral areas of vasogenic edema in the parieto-occipital lobes and cerebellum consistent with PRES. An incidental subacute spinal subdural hematoma extending from the level of C6 to T1 was depicted by spinal magnetic resonance imaging, presumably as a complication of negligible neck trauma. Spinal digital subtraction angiography showed no evidence of spinal aneurysm, arteriovenous malformation, or dural arteriovenous fistula. Cerebral digital subtraction angiography showed segmental narrowing and dilatation of vessels, a potential feature of RCVS, involving the circle of Willis and their branches.

**Interventions:**

The patient was treated with nimodipine for vasodilation and other symptomatic therapies. The spinal subdural hematoma was not warranted for surgical intervention and managed with simple analgesics.

**Outcomes:**

The patient experienced a dramatic improvement in neurological symptoms and was discharged without sequelae. Follow-up imaging showed complete resolution of all radiological changes.

**Lessons:**

Clinician should be aware of spinal subdural hematoma as the potential trigger in development of PRES and RCVS. We speculate that endothelial dysfunction and vascular tone dysregulation may be implicated to play the major pathophysiologic role.

## 1 Introduction

Posterior reversible encephalopathy syndrome (PRES) is a clinical radiographic syndrome of heterogeneous etiologies presented with varied neurological symptoms, which may include headache, altered level of consciousness, visual disturbance, and seizures.^[1,2]^ Neuroradiographic abnormalities of PRES are best depicted by magnetic resonance imaging (MRI), with bilateral reversible vasogenic edema in the parieto-occipital lobes as the typical feature.^[3]^ But lesions involve frontal lobes, cervical spinal cord or isolated posterior fossa regions are also described in the literature.^[3–5]^ A wide variety of medical conditions have been described related to PRES, with hypertensive crisis or emergency, eclampsia, renal failure, and use of cytotoxic and immunosuppressant drugs being the most common.^[1,5]^ Although the pathogenesis of PRES remains unclear, accumulating evidences suggest disordered cerebral autoregulation and endothelial dysfunction as the 2 possible pathophysiological explanations.^[6,7]^

Reversible cerebral vasoconstriction syndrome (RCVS) is another increasingly recognized vasculopathy characterized by diffuse segmental constriction of cerebral arteries that resolves spontaneously within 3 months. The clinical manifestations are always uniphasic, which vary from pure cephalalgic forms to rare catastrophic forms associated with several hemorrhagic and ischemic strokes.^[8,9]^ More than half the cases occur in the context of postpartum or after exposure to adrenergic or serotonergic drugs.^[8,10,11]^ Further conditions related to RCVS are history of migraine, unruptured saccular aneurysms, the neurosurgical procedures, and sexual activity.

Though less commonly, the presence of reversible lesions indicating transient brain edema in patients with RCVS and multifocal cerebral vasoconstriction noted in patients with PRES whenever angiography included were increasingly documented, suggesting an overlapping pathophysiology between RCVS and PRES.^[12,13]^ Here we report a unique case of 40-year-old female presented with both RCVS and PRES associated with spinal subdural hematoma. We hypothesize that the incidental spinal subdural hematoma due to neck trauma may act as the trigger in development of RCVS and PRES, which resulted in severe clinical manifestation.

## 2 Case presentation

A 40-year-old woman was admitted to our emergency department after a series of generalized tonic-clonic seizure. Two days before the visit, she complained of crescendo headache associated with nausea and vomiting. She had a minor neck injury 1 week ago but attracted no more attention. Her past medical history revealed previous diagnosis with hypertension for 10 years and she was on irregular amlodipine treatment. Her family history was negative for any neurological disorder and she did not have any history of diabetes or smoking.

On admission, the patient was somnolent and lagged in response to external stimulation. Her abilities to understand, recall, and orient herself had significantly diminished. Neurological tests yielded a Glasgow Coma Scale score of 13. There was neck stiffness on neck jolt maneuver but with no other focal neurological deficits. Physical examination revealed body temperature of 38.3°C, heart rate of 84 beats per minute, respiration rate of 20 breaths per minute, and blood pressure of 160/92 mm Hg (1 mm Hg = 1.33 kPa).

The patient underwent conventional electroencephalogram and cranial computed tomography (CT) that were all normal. However, xanthochromia and hemorrhagic cerebrospinal fluid (CSF) with pleocytosis was found on subsequent lumbar puncture. The opening pressure of CSF was above 400 mmH2O and analysis of CSF composition showed 158 white blood cells/μL, 140000 red blood cells/μL, protein level 1.470 g/L, and glucose level 1.0 mmol/dL, respectively. CSF mycobacterium PCR, cryptococcal antigen test, and ink stain were negative. Complete blood count, glucose, electrolytes, coagulation studies, assessment of hepatic, renal and thyroid functions were unremarkable only with mild thrombocytopenia (Table 1).

**Table 1:**
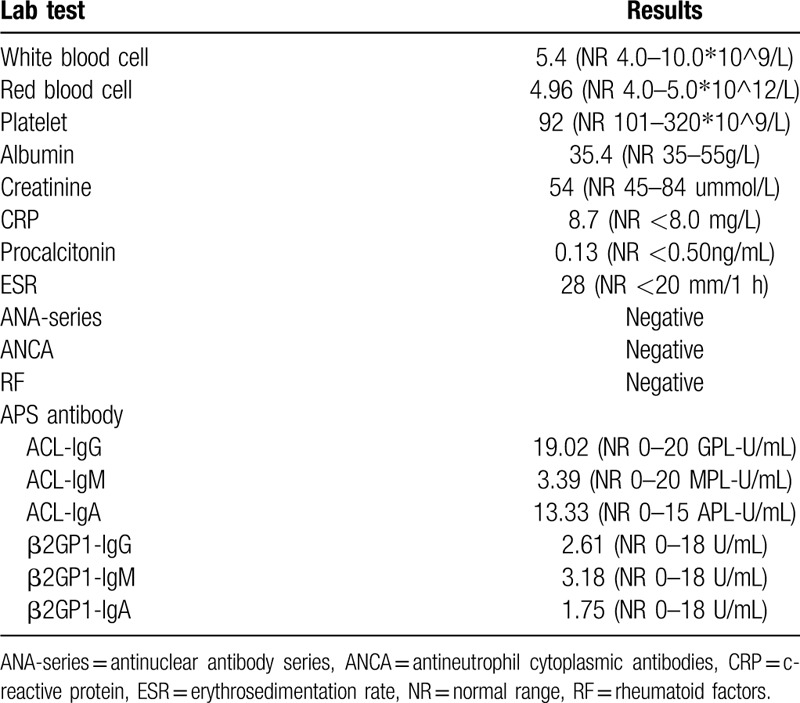
Laboratory findings of the patient.

Considering the inflammatory and hemorrhagic CSF results in the clinical setting of fever and positive meningeal irritation sign, initial working diagnosis was made with suspect of intracranial infection and subarachnoid hemorrhage. Search for common etiologies of subarachnoid hemorrhage such as aneurysm or vascular malformation on cerebral CT angiography (CTA) was negative but the results demonstrated multiple arteries narrowing. Empirical antibiotic treatment with ceftriaxone based on experience was immediately initiated but terminated within 24 hours due to severe allergic response. Other symptomatic treatments during the hospitalization included temporary application of mannitol for intracranial pressure reduction, urapidil for blood pressure control, and anticonvulsant therapy with levetiracetam.

**Figure 1:**
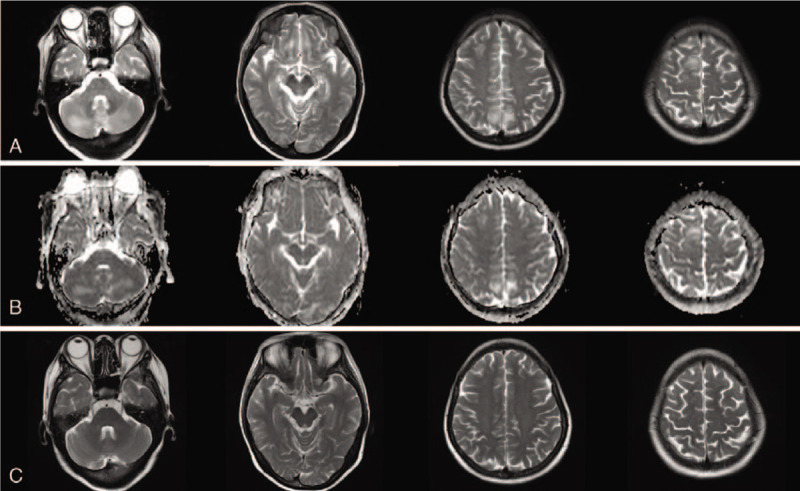
Initial and follow-up cranial MRI. (A, B) Initial cranial MRI on 3rd day after admission showed extensive vasogenic edema in bilateral parieto-occipital lobes and cerebellum. (C) One-month follow-up cranial MRI showed complete resolution of the edematous lesions. MRI = magnetic resonance imaging.

**Figure 2:**
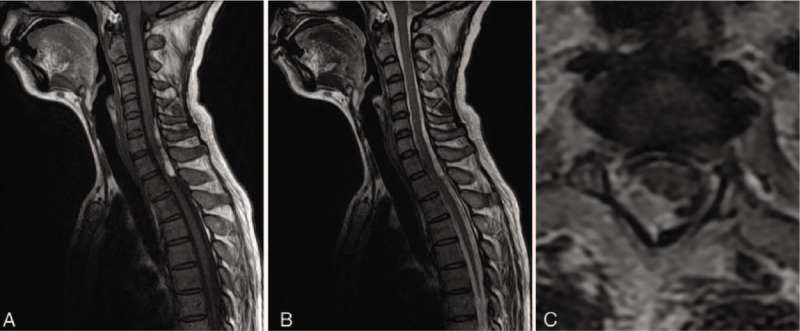
Spinal MRI obtained on day 7 during hospitalization. (A, B) Sagittal spinal MRI showed a subacute subdural hematoma extending from the level of C6 to T1. (C) T2-weighted transversal image at the level of C8 showed the recent hemorrhage in subdural space with no obvious thecal sac compression. MRI = magnetic resonance imaging.

**Figure 3:**
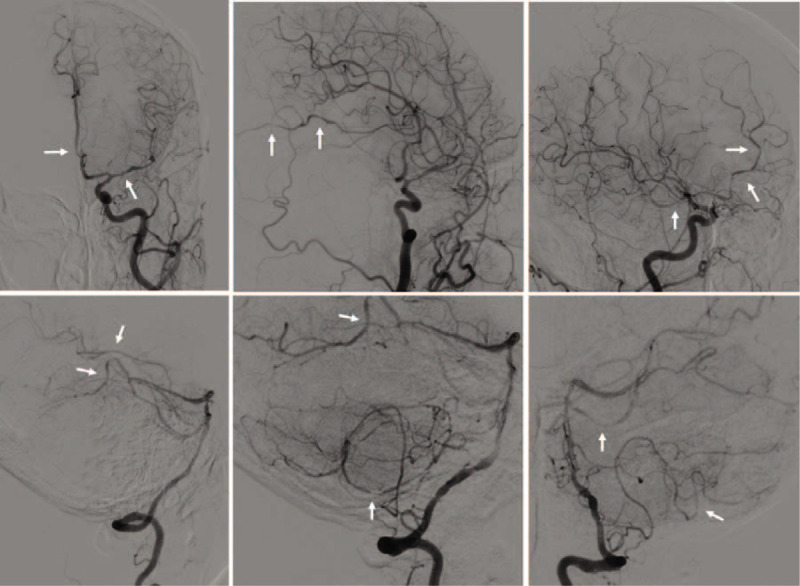
Cerebral digital subtraction angiography (DSA) showed multiple segmental narrowing and dilatation of the intracranial arteries involving both anterior and posterior circulation (arrows).

On the 3rd day of hospitalization, the patient's consciousness returned to normal and repeated evaluation by Glasgow Coma Scale score was 15. She was able to communicate normally and complained of blurred vision, headache, and neck tenderness. Cranial MRI on the same day revealed bilateral vasogenic edema in cerebellum as well as parietal-occipital lobes, which was interpreted as typical PRES (Fig. 1). The rapid recovery of symptoms on the discontinuation of antibiotic therapy and unsupported imaging features made the presumptive intracranial infection unreliable. In order to elucidate the abnormality in CSF and assess any comorbidity that may contributed to the development of PRES, the patient was transferred to the department of neurology ward for further evaluation. Repeated lumbar puncture on day 5 revealed a tendency of normalization in CSF cell counts and biochemistry (Table 2). As the clinical symptoms of the patient had significantly improved, the previous stated but neglected neck trauma had regained its attention. We performed the spinal MRI on day 7 that showed a trip of subacute hematoma in subdural space extending from the level of C6 to T1, which was considered accompanied with spinal subarachnoid hemorrhage and explained hemorrhagic CSF results (Fig. 2). Cerebral and spinal digital subtraction angiography (DSA) was subsequently performed to reveal any structural abnormality. In comparison with previous CTA, the cerebral DSA showed multiple segmental narrowing and dilatation of vessels (“string of beads” or “sausage on a string”), a potential feature of RCVS, involving the circle of Willis and their branches (Fig. 3). The spinal DSA showed no evidence of spinal aneurysm, arteriovenous malformation or dural arteriovenous fistula. Extensive laboratory examinations including erythrocyte sedimentation rate, rheumatoid factor, antinuclear and anti-neutrophil cytoplasmic antibodies and anticardiolipin antibodies were all normal. Chest CT, echocardiography, and abdominal ultrasonography were unremarkable (Table 1).

**Table 2:**
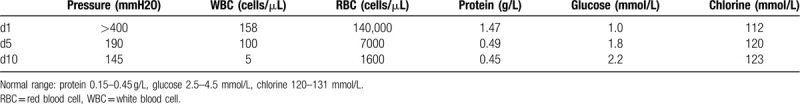
Analysis of cerebrospinal fluid.

As the spinal subdural hematoma was relatively small with no obvious thecal sac compression, surgical intervention was not warranted and her neck tenderness was managed with simple analgesics. Nimodipine was also added for vasodilation and seem to relieve headache successfully. Her symptoms gradually disappeared over 5 days and improvement in repeated CSF examination was observed (Table 2). The patient was discharged on day 10 without sequelae. One-month follow-up brain MRI showed complete resolution of the subcortical edematous lesions. Cerebral magnetic resonance angiography at 3 month demonstrated normal arterial appearance.

## 3 Discussion and conclusion

The combination of PRES and RCVS have already been described in females with hypertension, preeclampsia or eclampsia, autoimmune diseases, intracranial hypotension, and use of vasoactive or cytotoxic drugs.^[6,9,14–17]^ Neuroradiological abnormalities consistent with PRES were observed in 8% to 38% of RCVS patients,^[10–12,18]^ whereas some PRES patients would represent reversible segmental vasoconstriction of intracranial arteries.^[10,13]^ These shared radiological characteristics combined with similar therapeutic strategies and benign clinical evolution, reversible once the potential causes are identified and treated, suggest an overlapping pathophysiology between RCVS and PRES. Some authors raised hypothesis that PRES and RCVS are actually a continuum of reversible disorders of the cerebral vascular function with different expressions.^[9,10]^ In RCVS, the typical segmental constriction of arteries proximal to the Willis circle often occur after the peak of clinical symptoms and is easily demonstrated by vascular imaging (magnetic resonance, or computed tomographic angiography, or transcranial Doppler). While the dysregulation of distal arterial branches of the cerebrovascular tree, which might explain the extensive vasogenic edema, cannot be visualized appropriately by direct conventional imaging. Endothelial dysfunction caused by circulating endogenous or exogenous toxins could be another pathomechanism implicated both in RCVS and PRES. More recent view is that endothelial dysfunction was taken as the intermediate pathway of the cascade.^[15]^ Normal function of the vascular endothelium is the structural basis for preservation of vascular integrity, dysfunction of which would lead to capillary leakage and blood-brain barrier disruption thus triggering vasogenic edema.^[1,2,19]^ In addition, immunogenic and vasoconstrictive agents released by injured vascular endothelial cells are thought to mediate cerebral vasospasm, which interpret the co-occurrence of RCVS.

In our patient, bilateral areas of white matter edema in the posterior cerebral hemispheres were consistent with PRES and complete resolution of neuroradiological abnormality on follow-up imaging further confirmed the diagnosis. Although multiple segmental narrowing of cerebral arteries was clearly demonstrated by DSA, the diagnosis of RCVS seemed less convincing on the background of the “confusing” CSF changes. According to previous literature, slight abnormalities of CSF – cell counts in the CSF within range of 5 to 35 per μL and protein concentration no more than 100 mg/dL, were reported in 0% to 60% of patients of diagnosis of RCVS.^[10,11,18]^ Angiographic differentiation included atherosclerosis, infectious arteritis, vasculitis, and fibromuscular dysplasia should be considered. However, lack of evidence in laboratory examinations and reversibility of vessel appearance after nimodipine treatment make the diagnosis other than vasoconstriction least likely. In addition, the smoothly tapered narrowing followed by abnormal dilated branches of the cerebral arteries were against vasculitis, as the arterial narrowing in latter was much more irregular. Cerebral vasospasm after aneurysm subarachnoid hemorrhage is a well-described phenomenon defined as narrowing of the large and medium-sized intracranial arteries around the vicinity of the rupture aneurysm. The diffuse segmental constriction of cerebral vessels involving both anterior and posterior circulation but relatively localized spinal subdural hematoma in our patient might indicate a different pathophysiological process from vasospasm after aneurysmal subarachnoid hemorrhage. Thus, we made the diagnosis of RCVS with cautious despite the abnormal CSF results.

After an extensive search for other etiology, we consider it is the spinal subdural hematoma that precipitate the development of RCVS and PRES. Spinal subdural hematoma is an uncommon condition with paraparesis or paraplegia, sensory level and pain as the most frequent presentations. Previous literature had indicated anticoagulation, arteriovenous malformation, lumbar puncture, and trauma may be the etiology.^[20]^ However, neurological complications secondary to spinal subdural hematoma seem rare and have never be reported before.

The pathophysiology between spinal subdural hematoma and both RCVS and PRES are believed to related to abnormalities in cerebral vascular tone and endothelial dysfunction. Experimental studies have shown that blood components and blood breakdown products in CSF can induce endothelial dysfunction and increase permeability of microvascular barriers, on the basis of which extravasation of serum from the vascular lumen into brain parenchyma would cause vasogenic edema.^[21–23]^ Indeed, global vasogenic edema in white matter and deep gray matter, although mild only measured by elevated apparent diffusion coefficient, was reported in the subacute stage of subarachnoid hemorrhage.^[24]^ Furthermore, the cell-free hemoglobin in the CSF disrupted dilatory NO signaling and increased endothelin-1, a powerful vasoconstrictor peptide, in cerebral arteries network, which shifted vascular tone balance from dilation to constriction.^[25,26]^ Therefore, we hypothesize a similar mechanism in spinal subdural hematoma triggering both RCVS and PRES. As local neck pain occurred several days after minor neck trauma, a venous origin of the subdural hematoma was inferred based on this delay onset. Although the rupture veins might be compressed due to increased pressure in subdural space thus preventing hematoma extension; the accumulated blood breakdown products in spinal subdural and subarachnoid space untimely cause leukoencephalopathy and cerebral vasoconstriction via pathomechanism of endothelial dysfunction and vascular tone dysregulation.

In conclusion, clinician should be aware of spinal subdural hematoma as the potential trigger in development of PRES and RCVS, especially when combined with previous trauma. However, the question whether the quantity or velocity of hemorrhage in subdural and subarachnoid space is involved in the mechanism remains controversial and requires further investigations in elaborate animal studies.

## Author contributions

HFC designed the case report and wrote the manuscript. HFC and YY examined the patient. HFC and ZQX analyzed the neuroimages. All authors read and approved the final manuscript.

## References

[1] Fischer M Schmutzhard E Posterior reversible encephalopathy syndrome. J Neurol 2017;264:1608–16. 28054130 10.1007/s00415-016-8377-8 PMC5533845

[2] Fugate JE Rabinstein AA Posterior reversible encephalopathy syndrome: clinical and radiological manifestations, pathophysiology, and outstanding questions. Lancet Neurol 2015;14:914–25. 26184985 10.1016/S1474-4422(15)00111-8

[3] Ollivier M Bertrand A Clarencon F Neuroimaging features in posterior reversible encephalopathy syndrome: a pictorial review. J Neurol Sci 2017;373:188–200. 28131186 10.1016/j.jns.2016.12.007

[4] McKinney AM Jagadeesan BD Truwit CL Central-variant posterior reversible encephalopathy syndrome: brainstem or basal ganglia involvement lacking cortical or subcortical cerebral edema. AJR Am J Roentgenol 2013;201:631–8. 23971457 10.2214/AJR.12.9677

[5] Fugate JE Claassen DO Cloft HJ Posterior reversible encephalopathy syndrome: associated clinical and radiologic findings. Mayo Clin Proc 2010;85:427–32. 20435835 10.4065/mcp.2009.0590 PMC2861971

[6] Hinchey J Chaves C Appignani B A reversible posterior leukoencephalopathy syndrome. N Engl J Med 1996;334:494–500. 8559202 10.1056/NEJM199602223340803

[7] Covarrubias DJ Luetmer PH Campeau NG Posterior reversible encephalopathy syndrome: prognostic utility of quantitative diffusion-weighted MR images. AJNR Am J Neuroradiol 2002;23:1038–48. 12063238 PMC7976914

[8] Ducros A Wolff V The typical thunderclap headache of reversible cerebral vasoconstriction syndrome and its various triggers. Headache 2016;56:657–73. 27015869 10.1111/head.12797

[9] Ducros A Reversible cerebral vasoconstriction syndrome. Lancet Neurol 2012;11:906–17. 22995694 10.1016/S1474-4422(12)70135-7

[10] Ducros A Boukobza M Porcher R The clinical and radiological spectrum of reversible cerebral vasoconstriction syndrome. A prospective series of 67 patients. Brain 2007;130:3091–101. 18025032 10.1093/brain/awm256

[11] Singhal AB Hajj-Ali RA Topcuoglu MA Reversible cerebral vasoconstriction syndromes: analysis of 139 cases. Arch Neurol 2011;68:1005–12. 21482916 10.1001/archneurol.2011.68

[12] Chen SP Fuh JL Wang SJ Magnetic resonance angiography in reversible cerebral vasoconstriction syndromes. Ann Neurol 2010;67:648–56. 20437562 10.1002/ana.21951

[13] Schwartz R Mulkern R Vajapeyam S Catheter angiography, MR angiography, and MR perfusion in posterior reversible encephalopathy syndrome. AJNR Am J Neuroradiol 2009;30:E19. 19208904 10.3174/ajnr.A1285 PMC7051378

[14] Feil K Forbrig R Thaler FS Reversible cerebral vasoconstriction syndrome and posterior reversible encephalopathy syndrome associated with intracranial hypotension. Neurocrit Care 2017;26:103–8. 27848124 10.1007/s12028-016-0320-4

[15] Staykov D Schwab S Posterior reversible encephalopathy syndrome. J Intensive Care Med 2012;27:11–24. 21257628 10.1177/0885066610393634

[16] Lee WJ Yeon JY Jo KI Reversible cerebral vasoconstriction syndrome and posterior reversible encephalopathy syndrome presenting with deep intracerebral hemorrhage in young women. J Cerebrovasc Endovasc Neurosurg 2015;17:239–45. 26523259 10.7461/jcen.2015.17.3.239 PMC4626349

[17] Tanaka K Matsushima M Matsuzawa Y Antepartum reversible cerebral vasoconstriction syndrome with pre-eclampsia and reversible posterior leukoencephalopathy. J Obstet Gynaecol Res 2015;41:1843–7. 26178813 10.1111/jog.12788

[18] Ducros A Fiedler U Porcher R Hemorrhagic manifestations of reversible cerebral vasoconstriction syndrome: frequency, features, and risk factors. Stroke 2010;41:2505–11. 20884871 10.1161/STROKEAHA.109.572313

[19] Marra A Vargas M Striano P Posterior reversible encephalopathy syndrome: the endothelial hypotheses. Med Hypotheses 2014;82:619–22. 24613735 10.1016/j.mehy.2014.02.022

[20] de Beer MH Eysink Smeets MM Koppen H Spontaneous spinal subdural hematoma. Neurologist 2017;22:34–9. 28009771 10.1097/NRL.0000000000000100

[21] Peeyush Kumar T McBride DW Dash PK Endothelial cell dysfunction and injury in subarachnoid hemorrhage. Mol Neurobiol 2019;56:1992–2006. 29982982 10.1007/s12035-018-1213-7

[22] Fujii M Duris K Altay O Inhibition of Rho kinase by hydroxyfasudil attenuates brain edema after subarachnoid hemorrhage in rats. Neurochem Int 2012;60:327–33. 22226843 10.1016/j.neuint.2011.12.014 PMC3288616

[23] Keep RF Andjelkovic AV Xiang J Brain endothelial cell junctions after cerebral hemorrhage: Changes, mechanisms and therapeutic targets. J Cereb Blood Flow Metab 2018;38:1255–75. 29737222 10.1177/0271678X18774666 PMC6092767

[24] Liu Y Soppi V Mustonen T Subarachnoid hemorrhage in the subacute stage: elevated apparent diffusion coefficient in normal-appearing brain tissue after treatment. Radiology 2007;242:518–25. 17179395 10.1148/radiol.2422051698

[25] Ciurea AV Palade C Voinescu D Subarachnoid hemorrhage and cerebral vasospasm - literature review. J Med Life 2013;6:120–5. 23904869 PMC3725434

[26] Zimmermann M Seifert V Endothelin and subarachnoid hemorrhage: an overview. Neurosurgery 1998;43:863–75. 9766314 10.1097/00006123-199810000-00083

